# Green and facile approach for enhancing the inherent magnetic properties of carbon nanotubes for water treatment applications

**DOI:** 10.1371/journal.pone.0180636

**Published:** 2017-07-14

**Authors:** Mohamed Ateia, Christian Koch, Stanislav Jelavić, Ann Hirt, Jonathan Quinson, Chihiro Yoshimura, Matthew Johnson

**Affiliations:** 1 Department of Civil and Environmental Engineering, Tokyo Institute of Technology, Ookayama, Tokyo, Japan; 2 Department of Chemistry, University of Copenhagen, Universitetsparken 5, Copenhagen Ø, Denmark; 3 Nano-Science Center, Department of Chemistry, University of Copenhagen, Universitetsparken 5, Copenhagen Ø, Denmark; 4 ETH Zürich, Institute of Geophysics, Sonneggstrasse 5, Zürich, Switzerland; Institute of Materials Science, GERMANY

## Abstract

Current methods for preparing magnetic composites with carbon nanotubes (MCNT) commonly include extensive use of treatment with strong acids and result in massive losses of carbon nanotubes (CNTs). In this study we explore the potential of taking advantage of the inherent magnetic properties associated with the metal (alloy or oxide) incorporated in CNTs during their production. The as-received CNTs are refined by applying a permanent magnet to a suspension of CNTs to separate the high-magnetic fraction; the low-magnetic fraction is discarded with the solvent. The collected MCNTs were characterized by a suite of 10 diffraction and spectroscopic techniques. A key discovery is that metallic nano-clusters of Fe and/or Ni located in the interior cavities of the nanotubes give MCNTs their ferromagnetic character. After refinement using our method, the MCNTs show saturation magnetizations up to 10 times that of the as-received materials. In addition, we demonstrate the ability of these MCNTs to repeatedly remove atrazine from water in a cycle of dispersion into a water sample, adsorption of the atrazine onto the MCNTs, collection by magnetic attraction and regeneration by ethanol. The resulting MCNTs show high adsorption capacities (> 40 mg-atrazine/g), high magnetic response, and straightforward regeneration. The method presented here is simpler, faster, and substantially reduces chemical waste relative to current techniques and the resulting MCNTs are promising adsorbents for organic/chemical contaminants in environmental waters.

## Introduction

Over the past decade, carbon nanotubes (CNTs) have found diverse applications because of their remarkable mechanical, thermal and electrical properties [1]. More specifically, carbon nanotubes functionalized with magnetic particles have attracted researchers’ interest in many fields including environmental science and waste management [2–4]. Applications of ‘magnetic CNTs’ (MCNTs) include catalysis [5], biomedical imaging [6], supercapacitors[7], biomanipulation [8], drug delivery [9], data storage [10] and environmental remediation [11, 12]. In particular, attention has focused on CNTs adsorption characteristics, due to their extremely large ratio of surface area to mass relative to most natural materials (e.g., clays and hydroxide minerals) and synthetic materials (e.g., activated carbon) [13–15]. Further, it has been proposed that MCNTs could be used to reduce the cost of environmental remediation as they could be easily separated from a solution using magnets without the need for centrifugal separation or filtration [16].

MCNTs can be either endohedral, i.e., made by encapsulating magnetic nanoparticles, such as iron oxides, into the carbon nanotubes, or exohedral, i.e., made with methods such as electrochemical deposition to attach magnetic nanoparticles on the surface of carbon nanotubes [1, 17, 18]. Previous studies showed that CNTs produced by different methods (including chemical vapour deposition and laser ablation) contain metal impurities, mainly Fe, Ni and Co, inside the tubes, which are covered with layers of graphene sheets [19, 20]. Today, most studies reporting preparation of MCNTs (**S1 Table**), seem to have overlooked three important facts. First, commercial CNTs with metallic impurities can be inherently magnetic due to the presence of Fe, Ni and Co in their metallic form [19, 21, 22]. Second, metallic impurities are hard to remove, even with concentrated acids at high temperature [23–25]. Third, using strong acids in a treatment step to remove residual metals is destructive and results in low yields (20–55 wt.%) [19].

From an environmental perspective, most existing methods for synthesizing MCNTs share a number of factors: 1) the use of strong acid to purify the CNTs, 2) large consumption of chemicals, 3) limited mass loading of metal oxides, and 4) modifications that may alter the surface chemistry of CNTs [2]. Therefore, there is a need to develop methods that are more economical, less complicated and work intensive, and more in line with the goals of green chemistry. A number of attempts have been made to improve the synthesis of MCNTs, however, so far it has not been possible to reduce the number of preparation steps [26] or the treatment temperature [2, 27].

We address two issues in this study. First, we propose a novel and simple approach, with modest chemical and thermal requirements, to enhance the inherent magnetic properties of commercial CNTs from different manufacturers. The obtained MCNTs were characterized to elucidate the origin of magnetization using 10 different diffraction and spectroscopic techniques. Second, we test the effect of our proposed approach on the adsorption capacity of CNTs by demonstrating its adsorption property to remove atrazine, a widely-used herbicide, from water. The adsorption properties of MCNTs and CNTs were compared using atrazine as a test compound, and MCNTs were regenerated in multiple consecutive cycles.

## Materials and methods

### Materials

Multi-walled CNT samples were purchased from Wako pure chemicals, Japan (CNT-Wako: diameter, 3–20 nm; length, ∼10 μm), and from Chengdu Alpha Nano Technology Co., Chinese Academy of Sciences, China (CNT-Alpha: diameter, 30–50 nm; length, ∼20 μm). In this article, the raw materials (i.e., as-purchased CNTs) are abbreviated as CNT-Wako and CNT-Alpha, and the magnetically separated samples are denoted MCNT-Wako and MCNT-Alpha. Ethanol (Sigma-Aldrich Co., Denmark) was used in the solvation/separation step of MCNTs. A stock solution of atrazine (500 mg/L) was prepared by dissolving 50 mg of standard atrazine powder (Sigma-Aldrich Co., Japan) in 100 mL of LC/MS grade methanol (Sigma-Aldrich Co., Japan) and kept refrigerated (4°C). Standard solutions were prepared by diluting the stock solution in appropriate amounts of Milli-Q water.

### Approach

**Fig 1:**
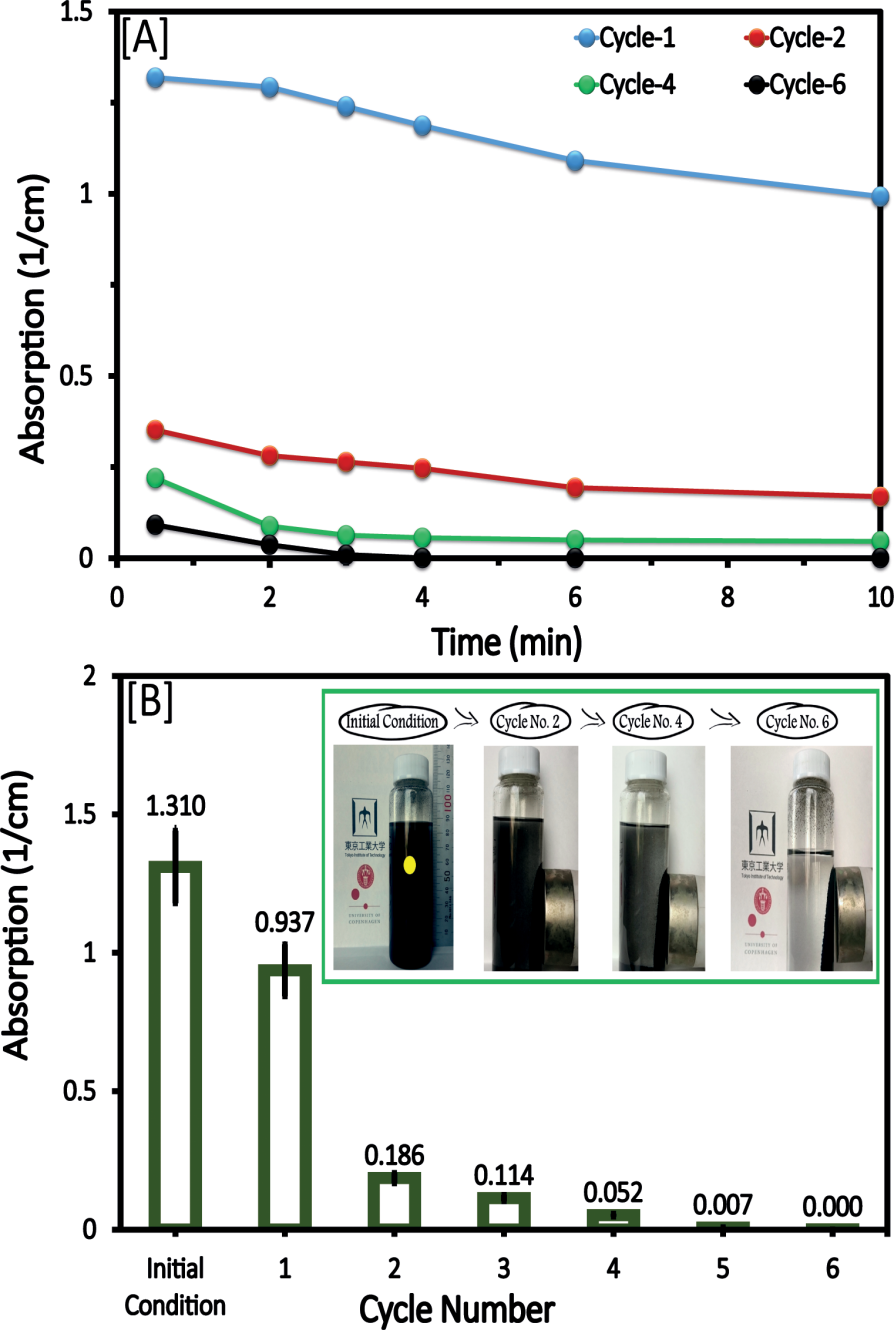
Absorbance at 800 nm of the supernatant (center of the vial) during the magnetic separation cycle. Panel [A] illustrates the MCNT collection efficiency as a function of time, and panel [B] shows the final residual amounts of magnetic CNT in the supernatant 10 min after introducing a permanent magnet adjacent to the suspension. Vertical lines are standard error bars from 3 replicates. Inset: Pictures taken at the end of the upgrading cycle (10 min after introducing the suspension to a permanent magnet). The yellow dot indicates the sampling point.

The refinement procedure was developed following the observation that a large fraction of the as-received CNTs were attracted to a permanent magnet, suggesting the possibility of collecting the magnetic fraction of as-received CNT samples from a liquid suspension. It was hypothesized that this method could separate MCNTs from amorphous carbon impurities and CNTs having small magnetic moments. To test, approximately 0.5 g of CNTs were dispersed in 100 mL of ethanol with a sonicator. Next, a permanent magnet (Nd-Fe-B MAGNET, Magna Co., Japan) was placed on the outside of the glass flask to attract the magnetic fraction and separate it from the non- and low-magnetic material. This procedure was repeated 3 or 4 times. Next, the same procedure was repeated using water as a solvent. The result of this procedure was a material that quickly responded to the magnetic separation procedure (Fig 1). The concentration of CNTs remaining in the supernatant was quantified by measuring the absorbance at 800 nm (Shimadzu UV-1800). As shown in Fig 1A, the time interval suitable for separating MCNTs is between 5 and 10 min. The efficiency of collection increased with the number of cleaning cycles (Fig 1B). The collected MCNTs were washed with water 3−4 times, dried and stored prior to use. It should be noted that: 1) the yield might include a small fraction of nonmagnetic CNTs that are associated with magnetic CNTs by electrostatic and/or chemical attraction [28], 2) The efficiency of the magnetic separation depends on many variables (e.g., aggregation state of the CNT and the amount and the distribution of the magnetic phases) [29], and 3) Ethanol was selected based on its ability to solvate and suspend the CNTs and its viscosity [30], which allowed us to maximize the separation efficiency by minimizing the possibility of forming CNT bundles [31].

### Characterization

The specific surface area was measured by the Brunauer−Emmett−Teller (BET) method. Pore volumes and pore size distributions were determined from nitrogen physisorption data at 77 °K obtained with ASAP 2020 analyzer (Micromeritics Instrument Corp., USA). Samples were prepared for TEM analysis by dispersing the solid powder of MCNT-Wako and MCNT-Alpha in ethanol and depositing a drop of the suspension on a carbon coated copper TEM grid. The instruments used were a Philips CM200 and a JEOL JEM-2100 both operated at 200 kV. Both microscopes were equipped with an EDAX detector for energy dispersive spectroscopy analysis (EDX).

Magnetic hysteresis was used to obtain information about particle size and composition. Analyses were carried out on small samples pressed into gelatin capsules using a Princeton Measurements Corporation (PCM) vibrating sample magnetometer (VSM). Hysteresis loops were measured using an averaging time of 200 ms, and a variable sample interval between 398 to 7958 A/m; the smallest sampling interval was used in fields < 160 kA/m, for an accurate determination of the coercivity. All magnetization results are normalized to the total weight of the sample. Due to the small sample size, it was not possible to determine the mass of the sample accurately, therefore saturation magnetization is only used to provide information on the order of the magnetization of the sample. First-order reversal curves (FORCs) were obtained by measuring 140 FORCs using 100 ms averaging time, by first saturating the magnetization in a field of 796 kA/m, before returning to the reversal field [32, 33]. The data were processed using FORCinel with a smoothing factor of 2, and the first point of each FORC was removed before processing [34]. This approach resulted in a diagram, which shows the spectra of coercivity of ferromagnetic particles in the sample on the horizontal axis (Hc), and interaction field (Hb) on the vertical axis. Magnetic susceptibility was measured as a function of temperature, i.e., thermomagnetic curves, using an AGICO MFK1_FA susceptibility bridge outfitted with a CS4 heating unit in order to determine Curie temperature of any ferromagnetic (*s*.*l*.) phases. The sample was heated between room temperature and 700°C in an Ar atmosphere, using a heating rate of 11°C/minute; the applied AC field was 200 A/m and 976 Hz.

X-ray photoelectron spectra (XPS) were collected using a Kratos Axis Ultra^DLD^ instrument. We used an AlKα monochromatic flux, with exciting radiation at 1486.6 eV and 150 W. The lens system of the photoelectron analyzer was set in the electrostatic mode to avoid rearranging and/or changing of the orientation of the magnetic particles during acquisition. Survey spectra were collected with the pass energy of 160 eV, and high-resolution spectra at 20 eV, with a resolution of 0.1 eV.

X-ray diffraction (XRD) diffractograms were acquired with Cu Kα_1.2_ (1.5406 Å), Ni-filtered radiation using a Bruker D8 Advance instrument equipped with a LynxEye PSD detector. Diffractograms were collected from 15–70° 2Θ with 0.03° 2Θ step size and 8 s time step, while the sample was spun at 15 rpm. The divergence slit was set to 0.3°, antiscatter slit to 3°, primary and secondary Soller slits were 2.5° and a detector window opening was 2.71°. The particle size of metal nanoparticles was determined from the XRD spectra by applying the standard Scherrer equation.

A Mössbauer spectrum of MCNT-Wako was measured at room temperature using a constant acceleration spectrometer. The very low concentration of Fe in the sample was partly compensated by using a thick sample, and partly by long measurement time. The spectrometer was calibrated using a thin foil of natural iron measured at room temperature. The hyperfine parameters of the spectral components were evaluated using a combination of sextet and singlet absorption lines having Lorentzian shape. The Fe content of sample MCNT-Alpha was too low for measuring a ^57^Fe Mössbauer spectrum. Raman spectra of all samples were recorded from 500 to 2500 cm^−1^ on NRS-4100 (JASCO) instrument using a 532 nm argon ion laser. JEOL JIR-SPX200 Japan was used to measure the FTIR spectra in the wavenumbers range of 250 to 4200 cm^−1^.

### Adsorption experiments

Adsorption experiments were performed in 40 mL glass bottles in a room with a temperature of 25±2°C. For each experiment, 40 mL of atrazine solution and the adsorbent (250 mg-CNT/L) were transferred to a series of bottles and 0.1 M HCl and/or 0.1 M NaOH were used to adjust the pH to 7 ± 0.2. Next the bottles were shaken using a regulated speed shaker under different substance doses and adsorption times.

The adsorption process was studied as a function of initial atrazine concentration (250 μg/L to 20 mg/L) with a contact time of 180 min (preliminary kinetic experiments are described in **S1 File**). After the adsorption, the samples were filtered through a membrane filter (0.45 μm PES filter, Membrane Solutions, Japan), and analyzed for atrazine concentrations. The adsorption capacity (*q*, mg/g) was obtained using the following equation (Eq 1):

$$q=\frac{C_{0}-C_{t}}{M}\times V$$ (1)

where *C*_*0*_ and *C*_*t*_ (mg/L) are the liquid phase adsorbate concentrations at the initial time and at a given time *t*, respectively, *V* is the experimental volume in liters and *M* is the adsorbent mass in grams.

The concentrations of atrazine were determined using HPLC (SHIMADZU Prominence UFLC, Japan) equipped with a pump (Shimadzu, model: LC-20AD) and a UV-Vis absorbance detector (SPD-20A UFLC), with a C18 Column, Kinetex 5 μm, 4.6 × 250 mm (Phenomenex CO., USA) operated with UV-Vis detection at 222 nm, a 100 μL injection volume and a 1 mL/min flow rate. All experiments were run in duplicate or triplicate, and the results are presented as the mean ± standard error.

Equilibrium isotherms are required to describe an adsorption system and estimate the adsorption characteristics of the adsorbent. To measure the isotherms, a known mass of the adsorbent is used and the concentration of the adsorbate varied over a certain range. The Langmuir (Eq 2), Freundlich (Eq 3), and Redlich–Peterson (RPM) (Eq 4) models were used to describe the adsorption of atrazine on both as-received CNTs and MCNTs [35].

$$q_{e}=\frac{q_{\max}bC_{e}}{1+bC_{e}}$$ (2)

where *q*_*e*_ (mg/g) is the adsorption capacity, *C*_*e*_ (mg/L) is the equilibrium concentration, *q*_*max*_ (mg/g) is the maximum capacity of adsorbate required to form a complete monolayer on the surface and *b* is the Langmuir constant. When *C*_*e*_*/q*_*e*_ is plotted against *C*_*e*_ and the data are regressed linearly, *q*_*max*_ and *b* constants can be calculated from the slope and intercept.

$$q_{e}=K_{F}C_{e}^{1/n}$$ (3)

where *q*_*e*_ and *C*_*e*_ are defined in the same manner as in the Langmuir equation. The Freundlich constant *K*_*F*_ is related to the adsorption capacity of the materials, and *1/n* is a constant related to surface heterogeneity. When log *q*_*e*_ is plotted against log *C*_*e*_ and the data are analyzed by linear regression, *1/n* and *K*_*F*_ constants are determined from the slope and intercept.

$$q_{e}=\frac{K_{R}C_{e}}{1+a_{R}C_{e}^{\beta }}$$ (4)

where *K*_*R*_ (L/g) and *a*_*R*_ (L/mg) are the RPM constants, and *β* is an exponent which lies between 0 and 1. When *β* = 1, it becomes a Langmuir equation; when *β* = 0, it becomes Henry’s Law.

### Regeneration experiments

MCNT-Wako and MCNT-Alpha were mixed with the atrazine solution in separate 100-mL glass bottles. After adsorption experiments, a permanent magnet was used to separate MCNTs and the supernatants were removed. Ethanol (10 mL) was added to MCNTs and the mixtures were oscillated for 10 min. The same process was repeated twice using ethanol and 3 times using water. The washed MCNTs were then recycled for adsorption. Initial conditions were atrazine concentration of 10 mg/L and MCNT concentration of 500 mg/L at pH 7 ± 0.2 and the equilibrium time at 180 min. We repeated these procedures ten times.

## Results and discussion

### Characterization of MCNTs

**Fig 2:**
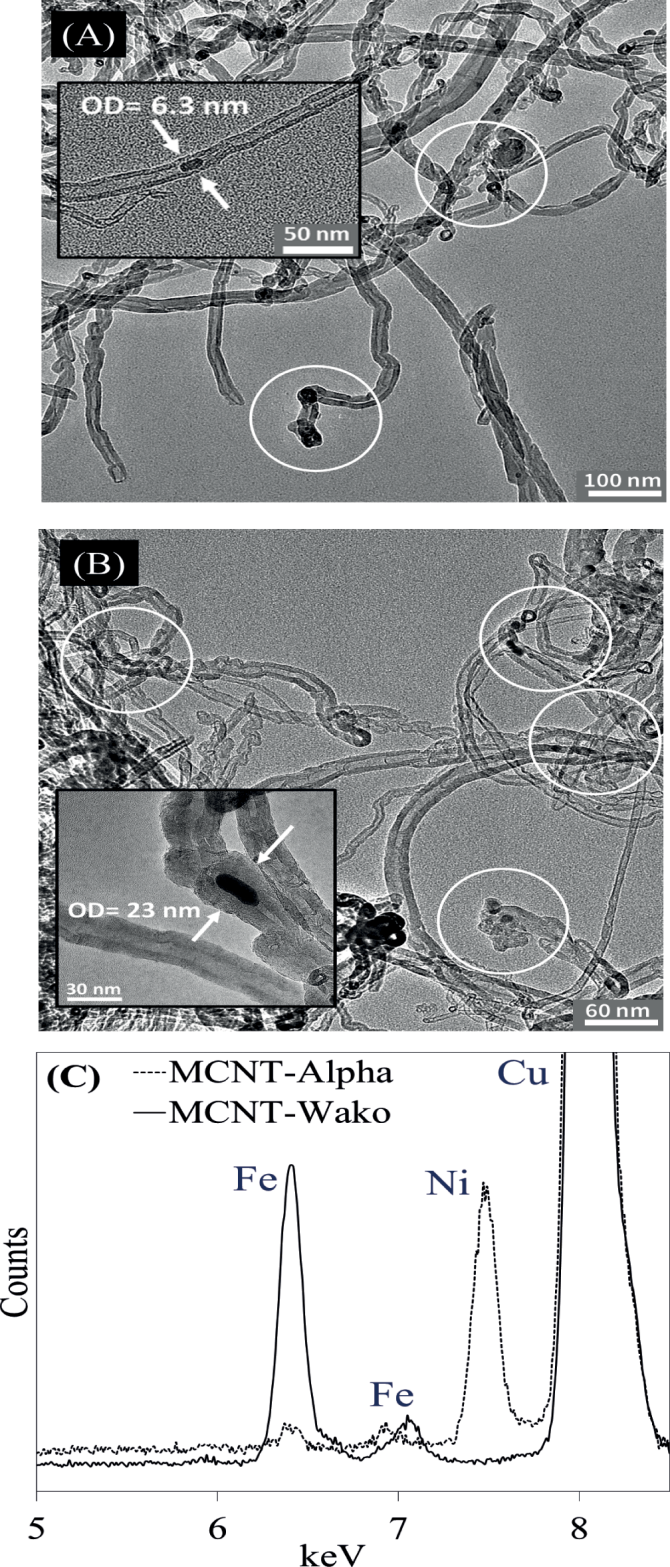
Low magnification micrographs of [A] MCNT-Wako and [B] MCNT-Alpha showing aggregates of CNT. The insets and white circles highlight the high-contrast particles containing metals within the CNT, and [C] EDX spectra for MCNT-Wako and MCNT-Alpha.

When the MCNTs are dispersed in water, a permanent magnet easily attracts the MCNTs and they can be collected on the vial’s wall within 5–10 min (Fig 1). The average yields (wt.%) from 4 replicates were similar for MCNT-Wako and MCNT-Alpha with calculated values of 96.4 ± 3.1% and 94.9 ± 2.6%, respectively. Table 1 summarizes the measured values for surface area, pore volume, and pore size distribution. Results show that surface area values for MCNT-Wako and MCNT-Alpha were as high as those for CNT-Wako and CNT-Alpha. Micrographs of the MCNT samples are shown in Fig 2A and 2B. The field of view is dominated by bundles that are frequently aggregated due to structural defects and/or van der Waals interactions between them [36], which was reflected on the increment of pore volumes (Table 1). Further, the tubes contain a tiny fraction of denser particles (insets of Fig 2A and 2B) [37, 38]. EDX analysis indicates that the samples contain Fe for MCNT-Wako and Ni with minor Fe for MCNT-Alpha (Fig 2C). Despite repeated attempts we did not succeed in obtaining electron diffraction spectra from the Fe and Ni rich particles to aid in their identification. Because Fe and Ni are the only identified elements that form magnetic phases, we infer that these particles give the aggregates a useful magnetic moment [23, 38, 39]. These observations were further confirmed in a series of experiments described in the following sections.

**Table 1:** Characteristics of as-received CNTs and magnetically separated MCNTs using our method.

Adsorbent	*SA*^a^	(*V*_*P*_)_*Total*_^a^	(*V*_*P*_)_*Micro*_^a^	(*V*_*P*_)_*Meso*_^a^	(*V*_*P*_)_*Macro*_^a^	OD
CNT-Wako	134	0.45	0.02	0.34	0.14	3-20^b^
CNT-Alpha	96	0.4	0.01	0.34	0.10	20-50^b^
MCNT-Wako	116	1.19	0.01	0.30	0.89	3–20
MCNT-Alpha	98	0.82	0.01	0.29	0.52	20–50

^a^ The BET surface areas, pore volumes and pore size distributions were measured from nitrogen physisorption data at 77 °K obtained with ASAP 2020 analyzer (Micromeritics Instrument Corp. U.S.). SA is surface area (m^2^/g), (*V*_*P*_)_*Total*_ is the total pore volume (cm^3^/g), (*V*_*P*_)_*Micro*_ is the volume of micropores (i.e., *pore* < 2 nm), (*V*_*P*_)_*Meso*_ is the volume of mesopores (i.e., 2 nm < *pore* < 50 nm), (*V*_*P*_)_*Macro*_ is the volume of macropores (i.e., *pore* > 50 nm). OD is outer diameter in nm. ^b^ Information provided by the suppliers.

**Fig 3:**
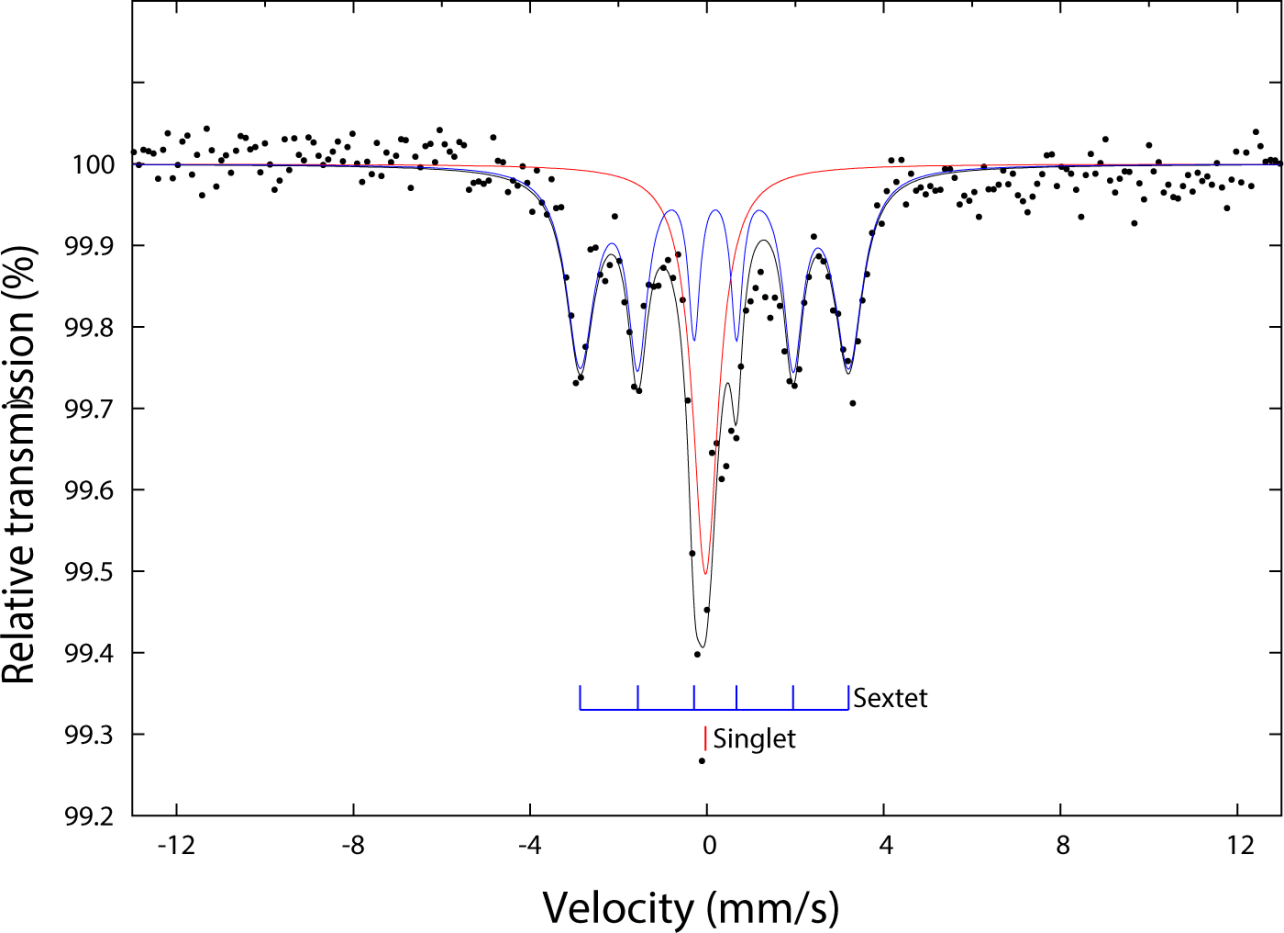
Mössbauer spectrum of MCNT-Wako at 293 K. The measurements are shown as black circles and the sum of the fitting components is shown as a black solid line. The peak positions (bar diagram) of the two fitting components (solid line in colours) are also shown.

The Mössbauer spectrum of the MCNT-Wako (Fig 3) sample was fitted using a sextet (magnetic hyperfine field of 18.8 T, an isomer shift of 0.18 mm/s, a negligible quadrupole shift, a linewidth of the outer lines of 0.8 mm/s, and a relative spectral area of 70%) and a singlet (isomer shift of 0 mm/s, line width of 0.7 mm/s, and relative spectral area of 30%). The parameters of the sextet are close to those reported for Fe_3_C (a catalyst used during CNT production [39, 40]). As in previous studies we observe a significant reduction in the magnetic hyperfine field and an increase in the line width in comparison to pure Fe_3_C [41, 42]. We interpret the deviation as being caused by extensive local disorder in the Fe_3_C particles of MCNT-Wako. The singlet is suggested to be due to the presence of superparamagnetic particles of Fe_3_C (see magnetic discussion). Fe_3_C is a ferromagnetic phase and although substitution and crystallographic defects may lower its magnetic moment, the results identify this phase as carrier of the magnetic properties in the Wako-MCNT.

**Fig 4:**
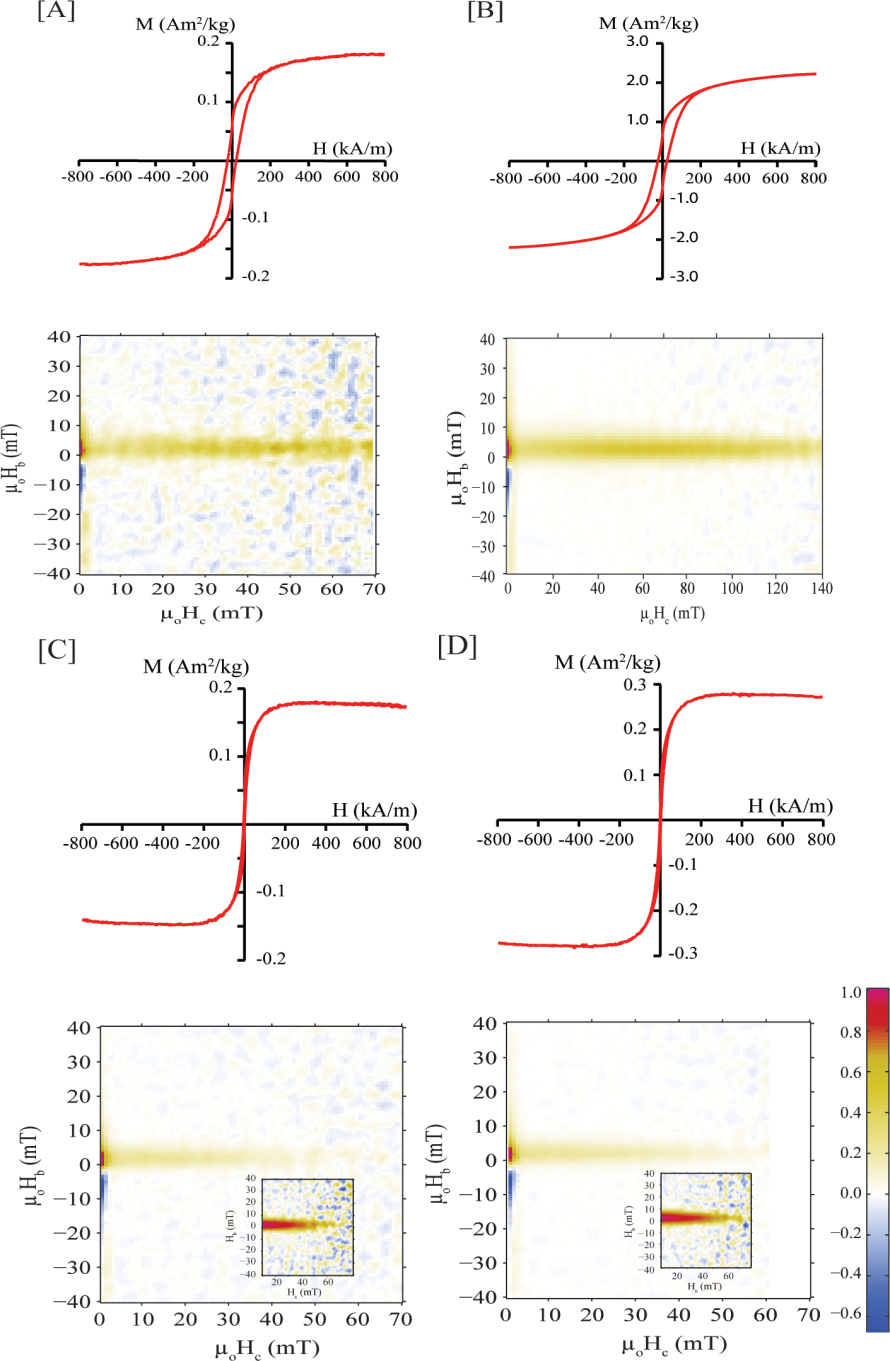
Magnetization curves up to 796 kA/m at 293 K and FORC diagrams for: [A] CNT-Wako, [B] MCNT-Wako, [C] CNT-Alpha, and [D] MCNT-Alpha. Inset in the FORC diagrams show the contribution of the higher coercivity for the Alpha samples.

Fig 4 shows the magnetization curves for CNT-Wako, MCNT-Wako, CNT-Alpha, and MCNT-Alpha. A weak diamagnetic component is seen in the hysteresis loop for CNT-Alpha and MCNT-Alpha. The saturation magnetizations of CNT-Wako and CNT-Alpha are similar. However, the saturation magnetizations for MCNT-Wako and MCNT-Alpha, after correcting for the high-field diamagnetic contribution in both Alpha samples; are 10 and 1.5 times the value of the as-received materials, respectively. The results show that our preparation method improves the magnetic activity of the material. It should be noted that using a weaker magnet during the upgrading process could result in further improvement in the magnetic property of the produced MCNTs. All loops are constricted, which suggests either the presence of two phases in which one phase has little to no coercivity, or two particle sizes of the same phase. Based on EDX, we conclude that there is only one phase but with varying size. The individual ferromagnetic particles are generally in a size range in which they should be superparamagnetic. Aggregation, however, can lead to clusters of larger size that act as single particles, i.e., larger effective particle size, that they carry a remanent magnetization and show coercivity.

FORC analysis was carried out to characterize the distribution of coercivity on the samples (Fig 4). All samples show a bimodal coercivity distribution. The dominant phase of the FORC distribution is at the origin, and is shifted upward on the Hb axis, which is indicative of superparamagnetic particles [33]. The second phase shows a broad coercivity distribution, which reflects contribution from the remanent carrying-phase that has varying particle size. Note that the low spread along Hb for all samples suggests little interaction between the magnetic particle clusters in the nanotubes.

**Fig 5:**
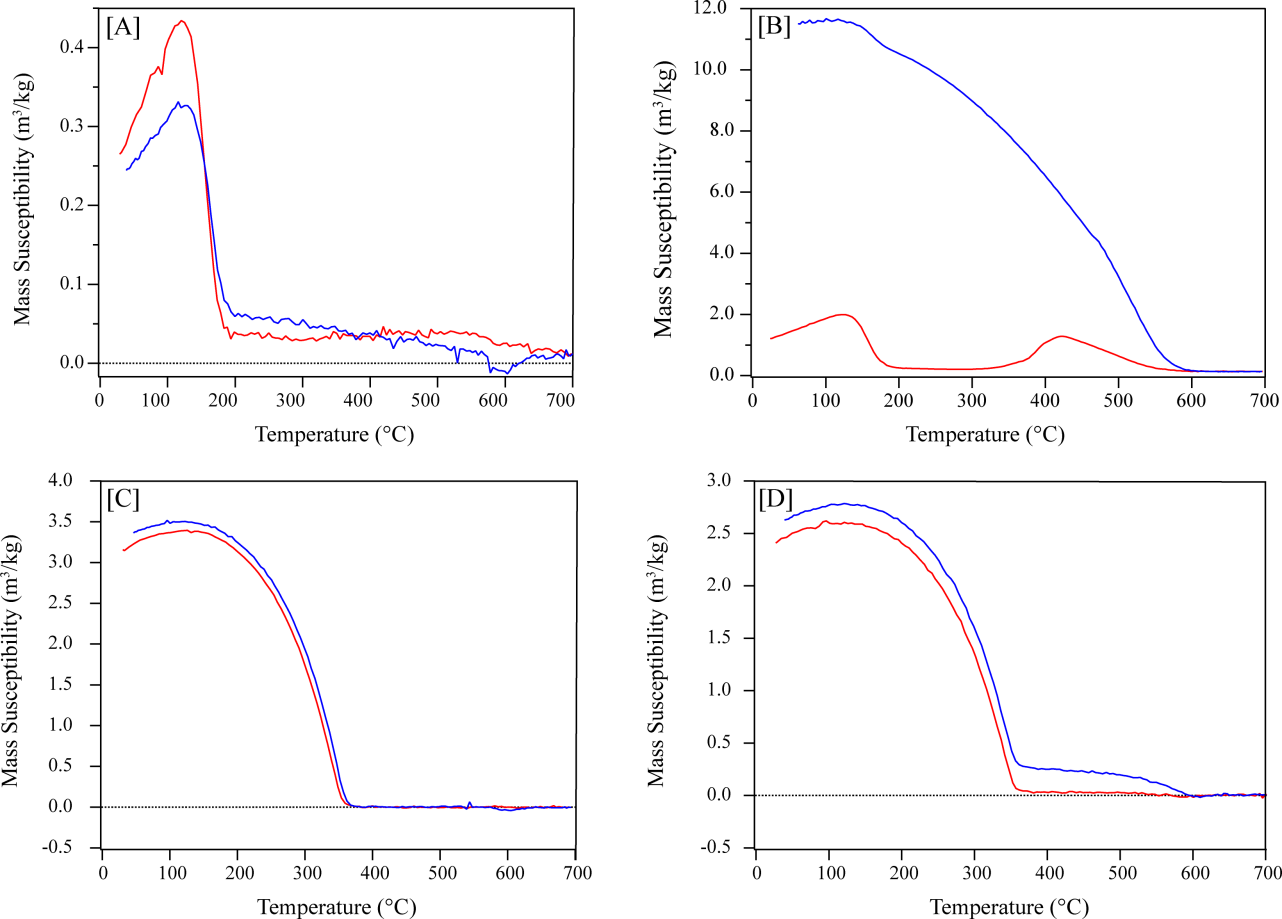
Thermomagnetic curves which illustrate the Curie temperature for: [A] CNT-Wako, [B] MCNT-Wako, [C] CNT-Alpha, and [D] MCNT-Alpha. Red/blue shows the heating/cooling curves, respectively.

The thermomagnetic curves for CNT-Wako and MCNT-Wako show a dominant magnetic phase at Curie temperature just under 200°C, and suggest higher temperature phases that may be related to magnetite (Fe_3_O_4_), and perhaps even higher temperature phases (Fig 5A and 5B). The lower temperature phases in CNT-Wako have a Curie temperature that is slightly lower than what is normally reported for Fe_3_C [43]. MCNT-Wako, however, shows high irreversibility and suggests the formation of new (modified) ferromagnetic phases with much stronger magnetization. Upon cooling, this phase dominates (Fig 5B). Based on the cooling curve the Curie temperature is around 580°C, which may indicate the formation of magnetite, or it may be due to the conversion of some Fe in the sample to magnetite during heating [44]. The lower temperature phase, however, is still present (i.e., the new formation is not at the expense of the original phase). As for CNT-Alpha and MCNT-Alpha, they show similar thermomagnetic curves, which are dominated by a phase having a Curie temperature around 340°C, which is consistent with the presence of Ni in the CNTs (Fig 5C and 5D). There is also an indication of a second phase with a Curie temperature around 580°C, which becomes more visible upon cooling in MCNT-Alpha. It must be noted that samples are stable under normal temperature conditions and changes are only observed during the extreme heating conditions used for phase identification of magnetic particles. Therefore, in terms of a stable inherent magnetization, the samples are suitable for applications in environmental remediation.

**Fig 6:**
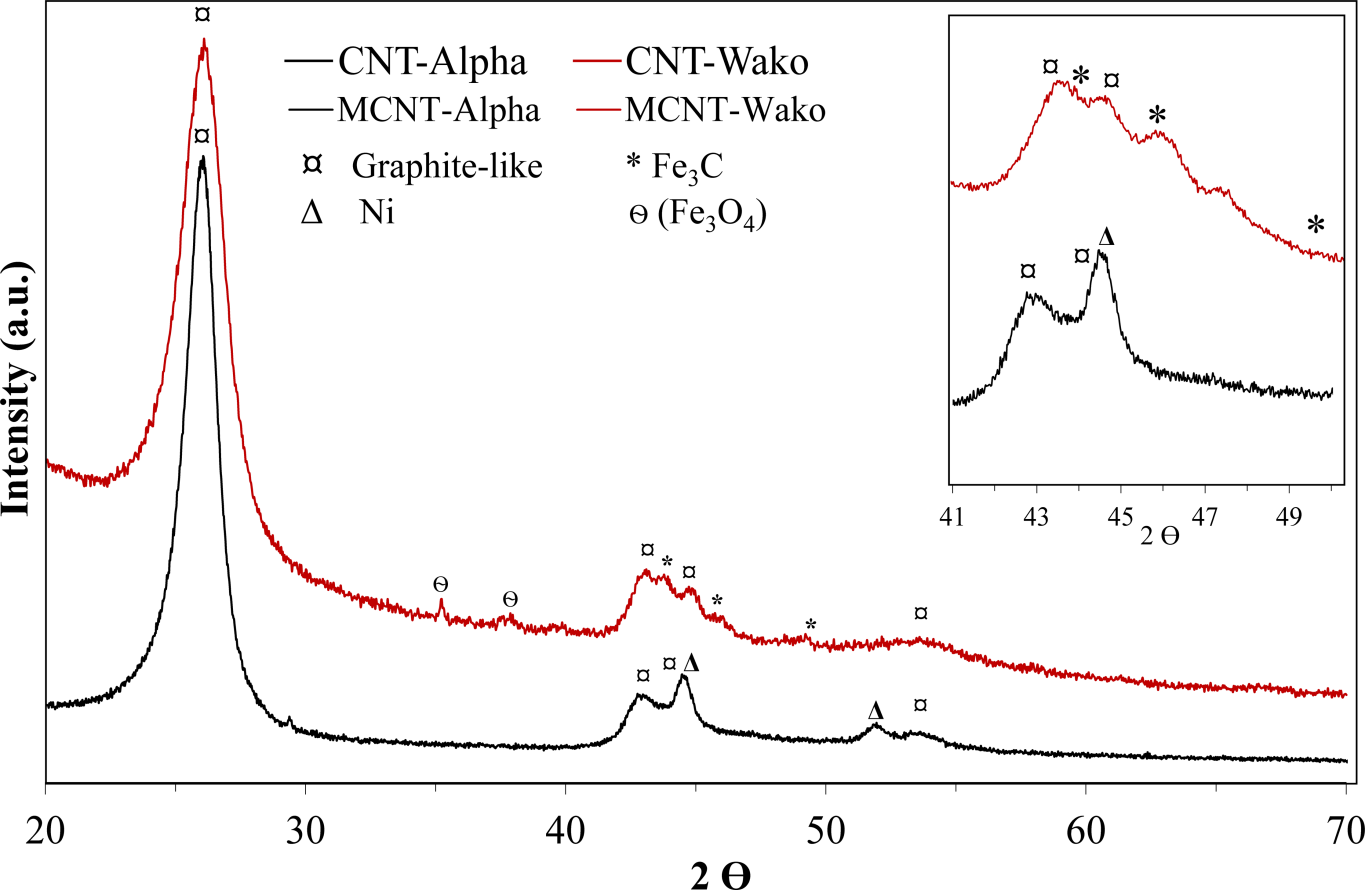
XRD traces of MCNT-Wako and MCNT-Alpha. The inset is enlargement of the range with most intense Fe/Ni diffraction peaks with a comparison among CNT-Wako, CNT-Alpha, MCNT-Wako and MCNT-Alpha.

Both samples show dominant, broad XRD diffraction peaks at ~26, 44 and 54 °2Θ that are attributed to diffraction from the graphite-like structure i.e. carbon nanotubes (Fig 6). In addition, MCNT-Wako shows a few broad peaks that can be assigned to Fe_3_C and magnetite (Fe_3_O_4_) [39, 45]. Thus, only a very few weak diffraction peaks remain unassigned. Magnetite is indicated by two minor peaks in agreement with the magnetic measurement but below the detection limit of Mössbauer spectroscopy. In the diffractogram from the MCNT-Alpha samples, two broad peaks can be assigned to Ni [37, 46], supporting the identification of this phase as the magnetic carrier of the MCNT-Alpha sample. The coherent scattering domain size of the Ni particles responsible for the magnetic response of the MCNT-Alpha sample is estimated using Scherrer’s equation, assuming a spherical domain shape (K = 0.93) and d = 9 nm. It is not possible to estimate the domain size of the MCNT-Wako sample as the amount of Fe-particles is too small for a reliable analysis.

In the surface analysis with XPS (**S2 Fig**), we only observe spectral lines from carbon and oxygen [37]. The explanation most commonly found in the literature focuses on the metal concentration, specifically that it might be below the detection limit of XPS [37–39]. However, from our observations, we suggest that the location of the metal nanoparticle is an equally important factor. Earlier studies by Edwards, et al., [38] and Antunes, et al., [39] detected peaks for Fe in CNT samples using XPS, however, CNTs in both studies contained metal impurities inside the tubes and inside the ‘tube walls’. XPS can probe only the few topmost nanometres of the material’s surface, suggesting that the iron and nickel phases are located within the carbon nanotubes and not on the surface or in the wall of MCNT-Wako and MCNT-Alpha. This conclusion is in agreement with the transmission electron microscopy (TEM) analysis.

**Fig 7:**
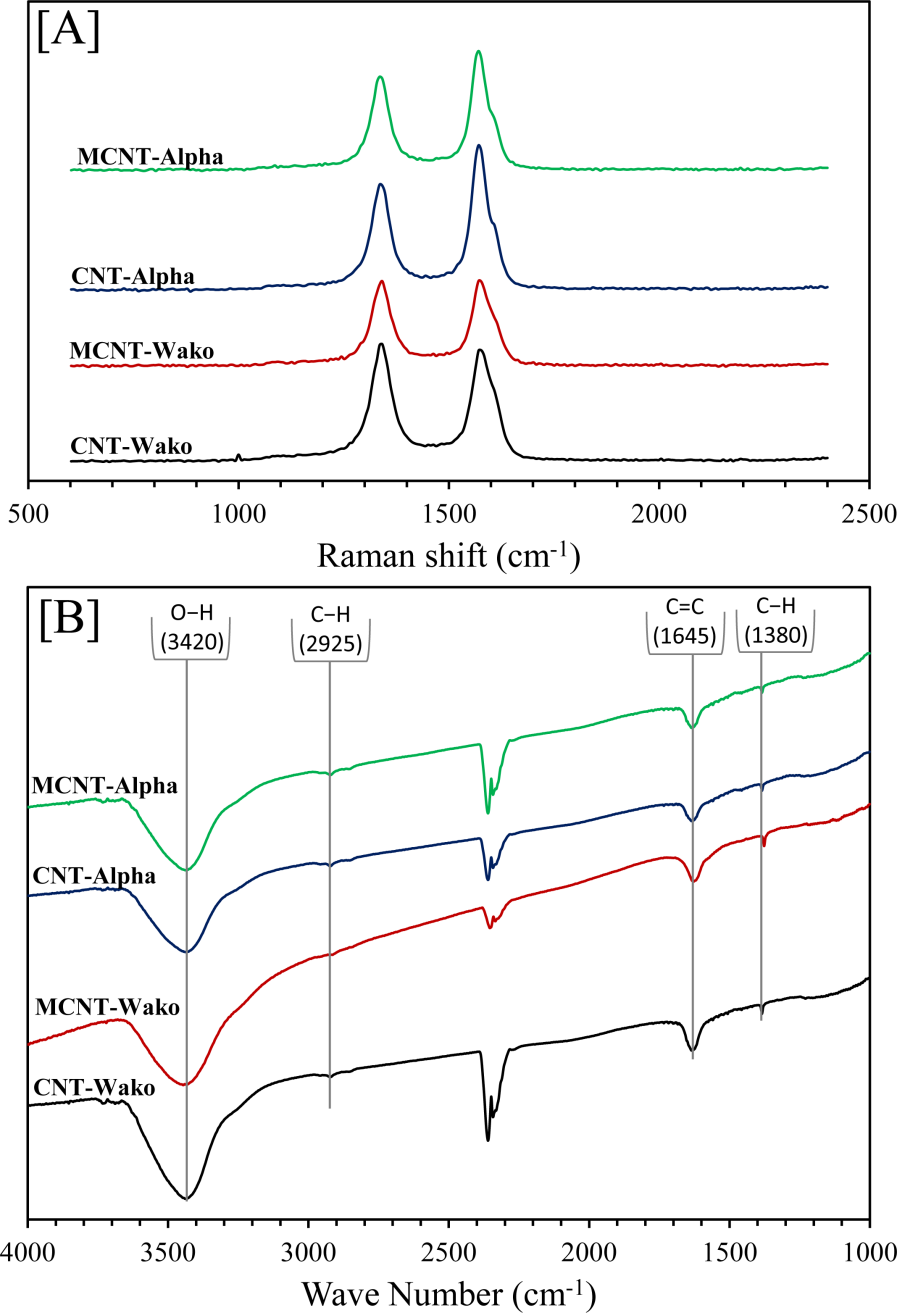
[A] The Raman spectra and [B] The FTIR spectra for CNT-Wako, MCNT-Wako, CNT-Alpha and MCNT-Alpha.

The Raman spectra of all samples are shown in Fig 7A. The peaks were observed at 1350 cm^−1^ for the disordered structure of CNT (D mode) which results from the E_2g_ phonon of sp^2^ atoms, and at 1590 cm^−1^ for the graphite structure of CNTs (G mode) which is a breathing mode of k-point phonons of A_1g_ symmetry. The intensity ratio (*I*_*D*_*/I*_*G*_) of the D band to the G band in graphitic materials was employed to determine the size of sp^2^ domains. The *I*_*D*_*/I*_*G*_ ratios of CNT-Wako, MCNT-Wako, CNT-Alpha and MCNT-Alpha are 1.05, 0.99, 0.88 and 0.93, respectively. The slight difference between *I*_*D*_*/I*_*G*_ ratio for Wako and Alpha samples suggests that the size of the sp2 domains is smaller for Alpha than Wako samples. However, there were no difference among as-received samples and the magnetic samples. This implies that our method does not alter the surface of CNT’s [46]. In addition, FTIR spectra showed similar four main broad bands for all samples at around 3420, 2925, 1645, and 1380 cm^−1^. These IR bands are ascribed to the stretching vibrations of O−H, C−H, C = C, and C−H bonds, respectively (Fig 7B) [47–49] and are also suggesting there is no change in the composition of CNT’s before and after the magnetic separation. The bands between 2200 cm^−1^ and 2400 cm^−1^ result from CO_2_ is in the beam path of the optics as a gas.

### Adsorption of atrazine

**Fig 8:**
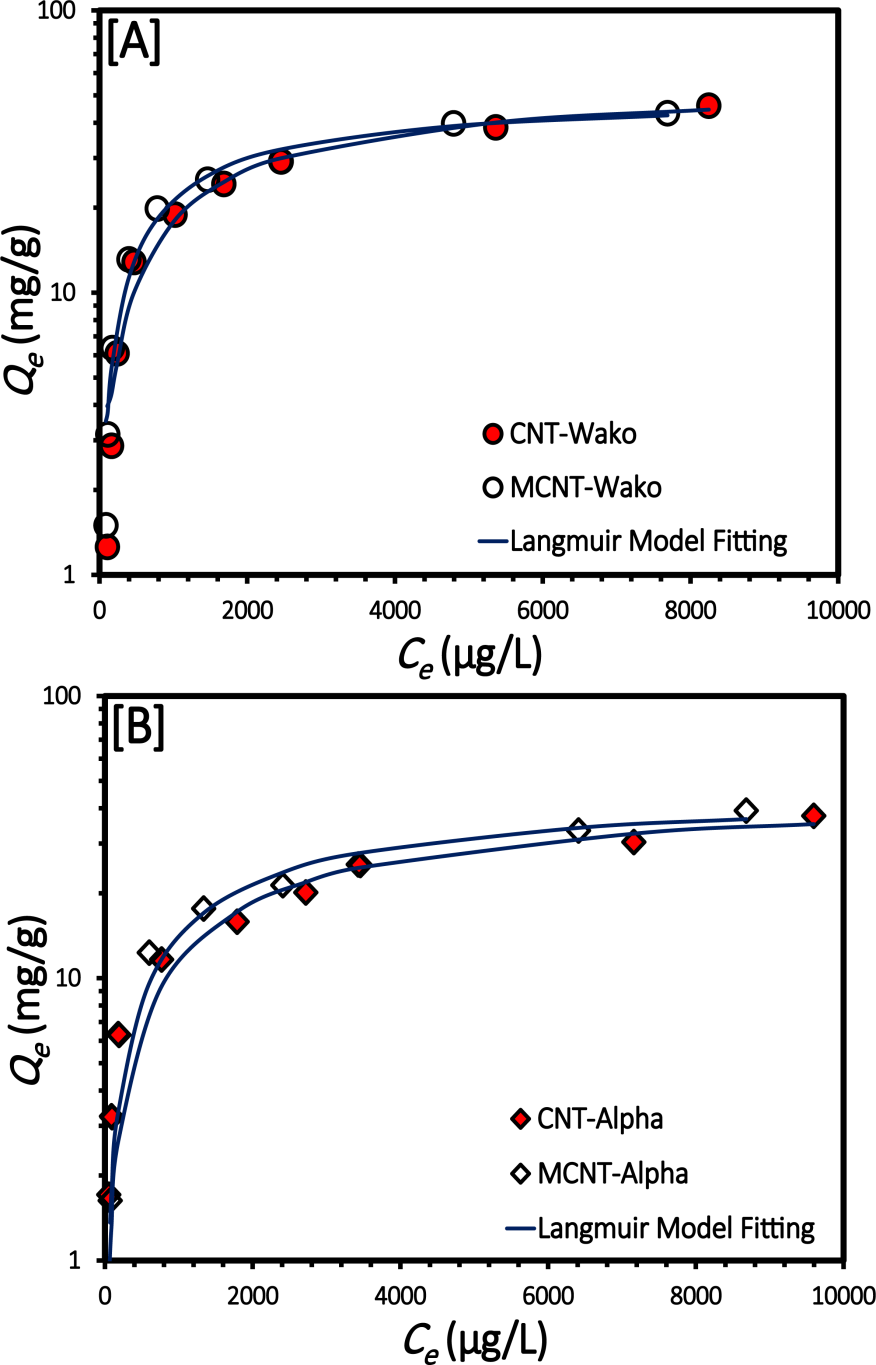
The adsorption isotherm of atrazine for [A] CNT-Wako and MCNT-Wako, and [B] CNT-Alpha and MCNT-Alpha.

Fig 8 shows the adsorption isotherms for atrazine on the CNT-Wako, CNT-Alpha, MCNT-Wako and MCNT-Alpha samples measured at 25±1°C, as the adsorbed amount of atrazine increases from the initial concentration. Adsorption isotherms describe the retention (or release) of material governing the mobility of a substance through an aqueous media to an adsorbent at a constant temperature and pH [35]. In the Langmuir isotherm we assumed that adsorption takes place at specific homogeneous sites within the adsorbent, that no significant interaction occurs among the adsorbed species, and that the adsorbent is saturated after one layer of adsorbate molecules forms on the adsorbent surface [47]. The values of *q*_*max*_ and *b* are presented in **Table 2**.

**Table 2:** Parameters of Langmuir, Freundlich, and Redlich-Peterson isotherms for atrazine adsorption on CNT-Alpha, CNT-Wako, MCNT-Alpha and MCNT-Wako.

Model	Parameters	Wako		Alpha	
		CNT	MCNT	CNT	MCNT
Langmuir isotherm	*q*_*max*_ (mg/g)	55.5	49.8	43.5	44.0
	*b* (L/mg)	0.49	0.35	0.75	0.43
	*R*^*2*^	0.99	0.97	0.98	0.98
Freundlich isotherm	*K*_*F*_ (mg/g.(L/mg)^1/n^)	16.5	15.7	14.3	12.6
	*1/n*	0.50	0.48	0.50	0.50
	*R*^*2*^	0.96	0.98	0.99	0.99
Redlich-Perterson isotherm	*K*_*R*_ (L/mg)	31.0	52.9	212.8	382.4
	*a*_*R*_ ((L/mg)^1/β^)	0.73	1.59	14.18	29.57
	*β* (L/mg)	0.88	0.77	0.51	0.51
	*R*^*2*^	0.99	0.99	0.99	0.99

The Freundlich isotherm is commonly used to describe the adsorption characteristics of multilayer and heterogeneous surfaces [48]. As shown in **Table 2**, the values of *1/n* were between 0 and 1, confirming that the adsorption processes are favorable. However, the Freundlich isotherm has been criticized for lacking a fundamental thermodynamic basis (e.g., not approaching the Henry’s Law at vanishing concentrations) [49]. The RPM isotherm addresses these concerns by considering the limitations of the Langmuir and the Freundlich isotherms [50]. Overall, according to **Table 2**, the experimental data show excellent fits within the following isotherms order Redlich−Peterson > Langmuir > Freundlich, based on their coefficient of determination (R^2^) values. Our results are in line with previous studies of atrazine adsorption by CNTs (e.g., Tang, Zeng, et al., [51], Rambabu, et al., [52], and Yan, et al., [53]). In this study, our target was to evaluate the effect of our method to prepare MCNTs on their adsorption capacity. However, it should be noted that adsorption capacity of atrazine by CNTs would be influenced by: 1) low initial atrazine concentrations result in reducing adsorption capacity as indicated by isotherm studies, and 2) water chemistry (e.g., pH, ionic strength, and the presence of natural organic matter [54, 55]).

When the adsorption capacities were normalized using the surface area values, they fall within a narrow range (**S3 Fig**), verifying the surface adsorption mechanism. Furthermore, the rapid uptake of atrazine by MCNTs within the first 10 min demonstrates a high affinity between atrazine and the surface of MCNTs [53] (**S1 Fig**). The dimensions of the atrazine molecules are ca. 0.96 nm × 0.84 nm × ~0.30 nm [52], and thus, the atrazine molecules can diffuse into the interstitial mesopores and macropores of the MCNTs (Table 1) [51].

Key advantages to using MCNTs as adsorbents are their superior magnetic properties and a high surface to volume ratio. However, most of the reported methods in the literature have claimed to have chemically attached magnetic particles to the surfaces of CNTs. These aggressive synthesis methods adversely affect the surface area and change the surface chemistry of the material by making it less inert. Chemical modification of the surface of MCNTs makes the surface more vulnerable to chemical degradation during regeneration, relative to an unmodified CNTs [56–60]. Thus, most of studies are evaluated in batch modes o nly and less emphasis is given to continuous operation that resembles industrial application. An important finding of our study is that the surface of magnetically separated MCNT’s is not loaded with metal oxides. The spectroscopic analysis shows that the particles responsible for the magnetic response are in the interior of tubes, sequestered from degradation as the material is recycled. Thus, the adsorption capacity and collection efficiency of our MCNTs, as prepared in this work, are not degraded with time as in other synthesis methods reported in the literature [61–63]. This finding suggests that MCNT’s with internally decorated magnetic particles show great potential for use in large scale processes because they are easily recycled.

### Regeneration of MCNTs

**Fig 9:**
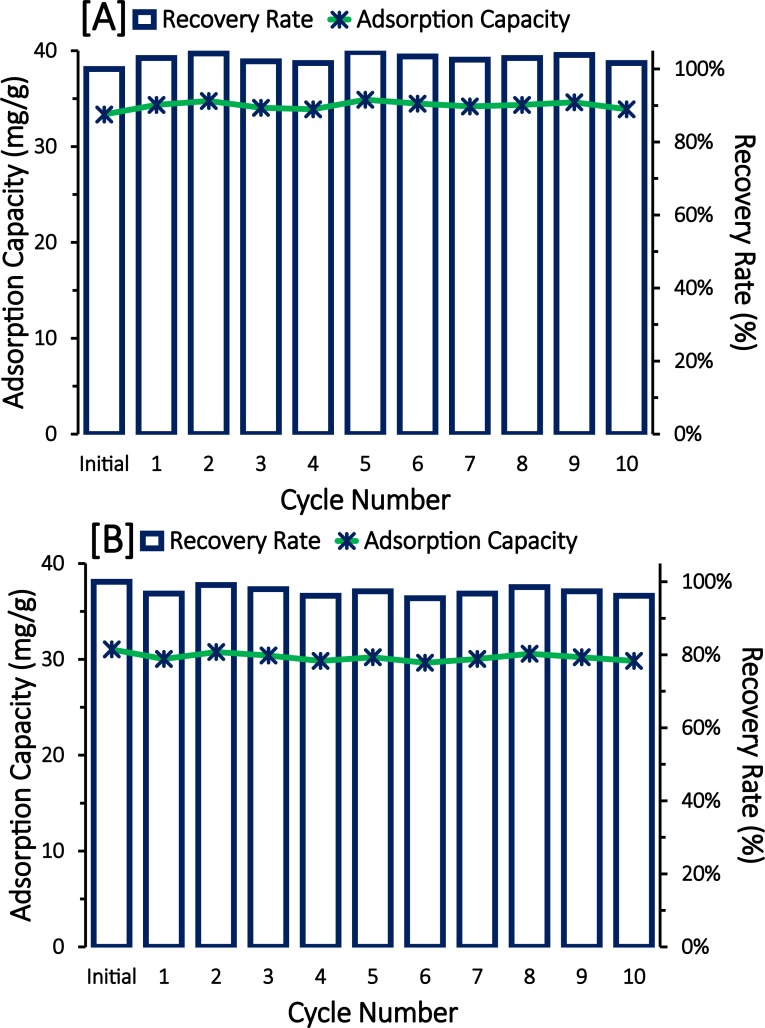
Adsorption capacity and recovery rate for [A] MCNT-Wako, and [B] MCNT-Alpha after ten reuse cycles. Initial atrazine concentration was 5 mg/L and MCNT concentration was 500 mg/L at pH 7 ± 0.2 and the equilibrium time at 180 min.

Chemical stability and recovery of the adsorption capacity are essential considerations for real-world applications of MCNTs to water treatment. In this study, both types of MCNT achieved 98 to 104% recovery rates tested over ten regeneration cycles. At the end of the experiment, the adsorbents weight was measured and showed negligible changes. As shown in Fig 9, the prepared MCNTs possessed good reusability, chemical inertness and collection efficiency in 10 consecutive cycles for efficiently removing atrazine from aqueous solution. Therefore, MCNTs are promising adsorbents and magnetic separators for organic/chemical contaminants in environmental water samples with high adsorption capabilities, high magnetic responses, and easy regeneration.

## Conclusions

This study shows a simple approach to utilize the inherited magnetic properties of CNTs to obtain magnetic CNTs. The approach consists of simple magnetic fractionation of commercial CNT’s. By eliminating commonly used acid treatment, our approach is considered to be environmentally friendly, scalable and inexpensive. This method upgrades the CNT without affecting their superior surface area and chemical composition of external nanotube walls. The resulting MCNT fractions shows saturation magnetization up to 10 times the value of the as-received materials. We employed these MCNTs to repeatedly remove atrazine from water in a cycle of dispersion into a water sample, collection by magnetic attraction, and regeneration by ethanol. In addition, the separated nonmagnetic fraction of CNTs can be used in applications that require minimal metal content (e.g., electrochemical applications, or in reactions that are sensitive to the presence of metal oxides).The resulting MCNTs show high magnetic response, high adsorption capacities (> 40 mg/g) as described well with Langmuir, Freundlich and RPM isotherms and straightforward regeneration. The adsorption properties of CNT’s did not change even after 10 times of recycling. It is also recommended that sorption should be tested in continuous-flow reactor systems with magnetic separation, regeneration, and recycling of MCNTs as adsorbents. On the whole, this method of producing MCNTs is greener compared to methods reported in literature and results in materials with superior characteristics that will contribute to reducing the cost for water treatment applications.

## Supporting information

S1 File Kinetics of adsorption. (PDF)

S1 Fig Adsorbed atrazine on MCNT-Wako as a function of equilibration time at two different initial concentrations 500 μg/L and 5 mg/L. (PDF)

S2 Fig Selected high resolution XPS scans for MCNT-Wako and MCNT-Alpha. (PDF)

S3 Fig Normalized adsorption capacity of atrazine by surface area of each adsorbent. (PDF)

S1 Table Studies on the preparation of magnetic carbon nanotubes. (PDF)
