# Supplementary material for: Green and facile approach for enhancing the inherent magnetic properties of carbon nanotubes for water treatment applications
Source: PLoS One. 2017 Jul 14;12(7):e0180636. doi: 10.1371/journal.pone.0180636 (PMC5510820; doi:10.1371/journal.pone.0180636)
Supplement: S1 File — (PDF) [file pone.0180636.s001.pdf]

Supporting Information for

Green and Facile Approach for Enhancing the Inherent Magnetic Properties of Carbon Nanotubes for Water Treatment Application

Mohamed Ateia[1*], Christian Bender Koch[2], Stanislav Jelavić[3], Ann M. Hirt[4], Jonathan Quinson[3], Chihiro Yoshimura[1], and Matthew S. Johnson[2*]

[1] Department of Civil and Environmental Engineering, Tokyo Institute of Technology, 2-12-1-M1-4 Ookayama, Tokyo 152-8552, Japan

[2] Department of Chemistry, University of Copenhagen, Universitetsparken 5, DK-2100 Copenhagen Ø, Denmark.

[3] Nano-Science Center, Department of Chemistry, University of Copenhagen, Universitetsparken 5, DK-2100 Copenhagen Ø, Denmark

[4] Institute of Geophysics, ETH Zürich, Sonneggstrasse 5, CH-8092, Zürich, Switzerland.

* Corresponding authors:
mohamedateia1@gmail.com [M. Ateia]

Submitted to: *PLOS ONE*

S1: *Kinetics of adsorption*

Adsorption experiments were performed with MCNT-Wako in 40 mL bottles in a controlled room temperature of 25 ± 2 °C. For each experiment, 40 mL of MilliQ water and the adsorbent were transferred to a series of bottles and sonicated for 30 min. 0.1 M HCl or 0.1 M NaOH were used to adjust the pH to 7.0 ± 0.2. Next the bottles were shaken using a regulated speed shaker under different substance doses and adsorption times. Experiments were conducted with two different initial concentrations of atrazine, 0.5 and 5.0 mg/L. Samples were withdrawn and filtered through a membrane filter (0.45 µm PES filter, Membrane Solutions, Japan).

The amount of adsorbed atrazine at time t (q_t, mg/g) was obtained using the following equation:

$$q_t = [\frac{C_0 - C_t}{M}] \times V \quad (1)$$

where C_o and C_t (mg/L) are the liquid phase adsorbate concentrations at the initial time and at a given time t, respectively, V is the experimental volume expressed in liters, and M is the adsorbent mass expressed in grams.

Adsorption is a time-dependent process and therefore the rate of adsorption is critical for evaluating the adsorbents and in designing treatment systems. In this sense, the design, including the residence times, can be optimized using the kinetic constants [1]. The influence of contact time on the adsorptive properties of CNTs and MCNTs are shown in **Fig. S1**. The adsorption of atrazine showed a fast increase in the initial part and and reached equilibrium in less than 1 h which agrees with the short equilibrium times reported in a previous study [2].

References

[1] X. Wu, K. Hui, K.S. Hui, S. Lee, W. Zhou, R. Chen, D. Hwang, Y. Cho, Y. Son, Adsorption of basic yellow 87 from aqueous solution onto two different mesoporous adsorbents, Chemical Engineering Journal, 180 (2012) 91-98.

[2] N. Rambabu, C.A. Guzman, J. Soltan, V. Himabindu, Adsorption characteristics of atrazine on granulated activated carbon and carbon nanotubes, Chemical Engineering & Technology, 35 (2012) 272-280.
