# Supplementary material for: Green and facile approach for enhancing the inherent magnetic properties of carbon nanotubes for water treatment applications
Source: PLoS One. 2017 Jul 14;12(7):e0180636. doi: 10.1371/journal.pone.0180636 (PMC5510820; doi:10.1371/journal.pone.0180636)
Supplement: S2 Fig — (PDF) [file pone.0180636.s003.pdf]

Supporting Information for

Green and Facile Approach for Enhancing the Inherent Magnetic Properties of Carbon Nanotubes for Water Treatment Application

Mohamed Ateia[1*], Christian Bender Koch[2], Stanislav Jelavić[3], Ann M. Hirt[4], Jonathan Quinson[3], Chihiro Yoshimura[1], and Matthew S. Johnson[2*]

[1] Department of Civil and Environmental Engineering, Tokyo Institute of Technology, 2-12-1-M1-4 Ookayama, Tokyo 152-8552, Japan

[2] Department of Chemistry, University of Copenhagen, Universitetsparken 5, DK-2100 Copenhagen Ø, Denmark.

[3] Nano-Science Center, Department of Chemistry, University of Copenhagen, Universitetsparken 5, DK-2100 Copenhagen Ø, Denmark

[4] Institute of Geophysics, ETH Zürich, Sonneggstrasse 5, CH-8092, Zürich, Switzerland.

* Corresponding authors:
mohamedateia1@gmail.com [M. Ateia]

Submitted to: *PLOS ONE*

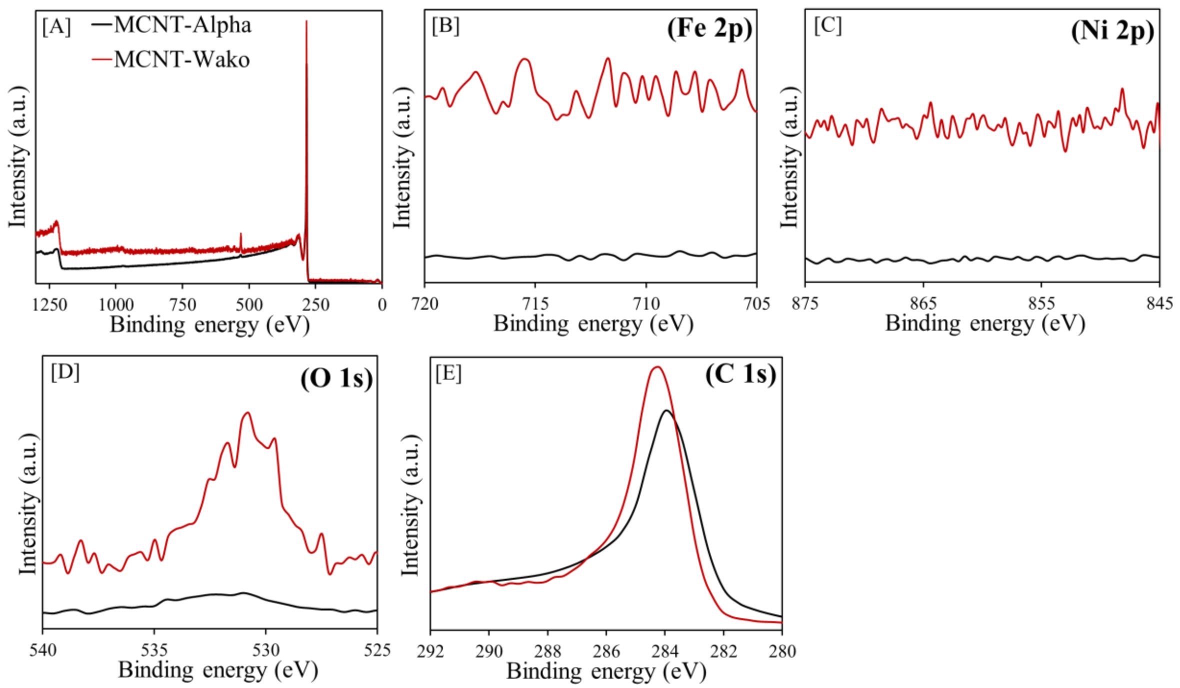

Fig. S2. [A] XPS scan for MCNT-Wako and MCNT-Alpha. Selected regions of high resolution scans for: [B] Fe 2p, [C] Ni 2p, [D] O 1s, and [E] C 1s spectra of MCNT-Wako and MCNT-Alpha. Only O 1s and C 1s peaks could be identified whereas, the Fe 2p and Ni 2p regions revealed only noise.
