# Supplementary material for: Green and facile approach for enhancing the inherent magnetic properties of carbon nanotubes for water treatment applications
Source: PLoS One. 2017 Jul 14;12(7):e0180636. doi: 10.1371/journal.pone.0180636 (PMC5510820; doi:10.1371/journal.pone.0180636)
Supplement: S1 Table — (PDF) [file pone.0180636.s005.pdf]

Supporting Information for

Green and Facile Approach for Enhancing the Inherent Magnetic Properties of Carbon Nanotubes for Water Treatment Application

Mohamed Ateia[1*], Christian Bender Koch[2], Stanislav Jelavić[3], Ann M. Hirt[4], Jonathan Quinson[3], Chihiro Yoshimura[1], and Matthew S. Johnson[2*]

[1] Department of Civil and Environmental Engineering, Tokyo Institute of Technology, 2-12-1-M1-4 Ookayama, Tokyo 152-8552, Japan

[2] Department of Chemistry, University of Copenhagen, Universitetsparken 5, DK-2100 Copenhagen Ø, Denmark.

[3] Nano-Science Center, Department of Chemistry, University of Copenhagen, Universitetsparken 5, DK-2100 Copenhagen Ø, Denmark

[4] Institute of Geophysics, ETH Zürich, Sonneggstrasse 5, CH-8092, Zürich, Switzerland.

* Corresponding authors:
mohamedateia1@gmail.com [M. Ateia]

Submitted to: *PLOS ONE*

Table S1. Studies on the preparation of magnetic carbon nanotubes.

Application	Acid Treatment	Acid treatment time (h)	Heat treatment (°C)	Test of magnetic properties after washing	Adsorption capacity reduction (%)*	Ref.
N.A.	Nitric Acid (14 M)	6	122	Not reported	N.M.	[1]
Drug delivery	Sulfuric acid and nitric acid mixture (mole ratio: 3/1)	12	80	Not reported	18-20	[2]
N.A.	Nitric Acid (15 M)	2	-	Not reported	N.M.	[3]
Solid-phase extraction (SPE)	Acetone	48	80	Not reported	N.M.#	[4]
N.A.	Nitric Acid (1 M)	6	20-25	Not reported	N.M.#	[5]
N.A.	Nitric Acid (65%)	3	130	Not reported $	N.M.	[6]
N.A.	-	-	-	Not reported	N.M.#	[7]
SPE	Nitric Acid (14 M)	8	60	Not reported	10-30	[8]
Adsorption of dyes	Sulfuric acid and nitric acid mixture (mole ratio: 3/1)	24 + 8	20-25	Not reported $	N.M.	[9]
SPE	Acetone	48	80	Not reported	N.M.#	[10]
SPE	Sulfuric acid and nitric acid mixture (mole ratio: 3/1)	6	20-25	Not reported	N.M.	[11]
N.A.	hydrofluoric acid and nitric acid	N.A.	N.A.	Yes	N.M.#	[12]
Adsorption of arsenic	12 M HCl	4	100	Not reported $	N.M.	[13]
N.A.	Nitric Acid (14 M)	6	140	Yes	N.M.#	[14]
Adsorption of aniline	-	-	-	Not reported $	N.M.#	[15]
N.A.	-	-	-	Not reported $	N.M.#	[16]
MIP for water treatment	Nitric Acid (2.6 M)	48	N.A.	Not reported $	N.M.#	[17]
MIP for water treatment	N.A.	N.A.	N.A.	Not reported	N.M.#	[18]
Adsorption of Co(II)	-	-	-	Not reported $	N.M.#	[19]
N.A.	-	-	-	Not reported $	N.M.	[20]
N.A.	Sulfuric acid and nitric acid mixture (mole ratio: 3/1)	6	80	Not reported $	N.M.#	[21]
Catalyst	Nitric Acid (8 M)	2	80	Not reported	N.M.#	[22]

N.M. = Not mentioned
* The reduction in the adsorption capacity after applying the proposed method to prepare magnetic carbon nanotubes.
# No reduction was mentioned, but TEM images indicate potential decrease of adsorption due to surface coverage with magnetic nanoparticles.
$ The authors claim that the metallic particles inside the tubes were introduced by their method.

References

[1] R. Liu, Y. Qiao, Y. Xu, X. Ma, Z. Li, A facile controlled in-situ synthesis of monodisperse magnetic carbon nanotubes nanocomposites using water-ethylene glycol mixed solvents, Journal of Alloys and Compounds, 657 (2016) 138-143.

[2] D. Xiao, P. Dramou, H. He, L.A. Pham-Huy, H. Li, Y. Yao, C. Pham-Huy, Magnetic carbon nanotubes: synthesis by a simple solvothermal process and application in magnetic targeted drug delivery system, Journal of Nanoparticle Research, 14 (2012) 1-12.

[3] F. Tan, X. Fan, G. Zhang, F. Zhang, Coating and filling of carbon nanotubes with homogeneous magnetic nanoparticles, Materials letters, 61 (2007) 1805-1808.

[4] A.V. Herrera-Herrera, J. Hernández-Borges, M.M. Afonso, J.A. Palenzuela, M.Á. Rodríguez-Delgado, Comparison between magnetic and non magnetic multi-walled carbon nanotubes-dispersive solid-phase extraction combined with ultra-high performance liquid chromatography for the determination of sulfonamide antibiotics in water samples, Talanta, 116 (2013) 695-703.

[5] Z. Sun, Z. Liu, Y. Wang, B. Han, J. Du, J. Zhang, Fabrication and characterization of magnetic carbon nanotube composites, Journal of Materials Chemistry, 15 (2005) 4497-4501.

[6] A. Demir, A. Baykal, H. Sözeri, R. Topkaya, Low temperature magnetic investigation of Fe 3 O 4 nanoparticles filled into multiwalled carbon nanotubes, Synthetic Metals, 187 (2014) 75-80.

[7] C. Gao, W. Li, H. Morimoto, Y. Nagaoka, T. Maekawa, Magnetic carbon nanotubes: synthesis by electrostatic self-assembly approach and application in biomanipulations, The Journal of Physical Chemistry B, 110 (2006) 7213-7220.

[8] D. Pardasani, P.K. Kanaujia, A.K. Purohit, A.R. Shrivastava, D. Dubey, Magnetic multi-walled carbon nanotubes assisted dispersive solid phase extraction of nerve agents and their markers from muddy water, Talanta, 86 (2011) 248-255.

[9] S. Qu, F. Huang, S. Yu, G. Chen, J. Kong, Magnetic removal of dyes from aqueous solution using multi-walled carbon nanotubes filled with Fe2O3 particles, Journal of Hazardous Materials, 160 (2008) 643-647.

[10] J. Ding, Q. Gao, X.S. Li, W. Huang, Z.G. Shi, Y.Q. Feng, Magnetic solid‐phase extraction based on magnetic carbon nanotube for the determination of estrogens in milk, Journal of separation science, 34 (2011) 2498-2504.

[11] N. Rastkari, R. Ahmadkhaniha, Magnetic solid-phase extraction based on magnetic multi-walled carbon nanotubes for the determination of phthalate monoesters in urine samples, Journal of Chromatography A, 1286 (2013) 22-28.

[12] C. Huiqun, Z. Meifang, L. Yaogang, Novel carbon nanotube iron oxide magnetic nanocomposites, Journal of magnetism and magnetic materials, 305 (2006) 321-324.

[13] B. Chen, Z. Zhu, J. Ma, M. Yang, J. Hong, X. Hu, Y. Qiu, J. Chen, One-pot, solid-phase synthesis of magnetic multiwalled carbon nanotube/iron oxide composites and their application in arsenic removal, Journal of Colloid and Ierface Sience, 434 (2014) 9-17.

[14] Y. Shan, K. Chen, X. Yu, L. Gao, Preparation and characterization of biocompatible magnetic carbon nanotubes, Applied Surface Science, 257 (2010) 362-366.

[15] M.A. Salam, M.A. Gabal, A.Y. Obaid, Preparation and characterization of magnetic multi-walled carbon nanotubes/ferrite nanocomposite and its application for the removal of aniline from aqueous solution, Synthetic Metals, 161 (2012) 2651-2658.

[16] J. Feng, J. Sui, W. Cai, J. Wan, A.N. Chakoli, Z. Gao, Preparation and characterization of magnetic multi-walled carbon nanotubes–poly (l-lactide) composite, Materials Science and Engineering: B, 150 (2008) 208-212.

[17] R. Gao, X. Su, X. He, L. Chen, Y. Zhang, Preparation and characterisation of core–shell CNTs@ MIPs nanocomposites and selective removal of estrone from water samples, Talanta, 83 (2011) 757-764.

[18] Y. Zhao, C. Bi, X. He, L. Chen, Y. Zhang, Preparation of molecularly imprinted polymers based on magnetic carbon nanotubes for determination of sulfamethoxazole in food samples, RSC Advances, 5 (2015) 70309-70318.
[19] Q. Wang, J. Li, C. Chen, X. Ren, J. Hu, X. Wang, Removal of cobalt from aqueous solution by magnetic multiwalled carbon nanotube/iron oxide composites, Chemical Engineering Journal, 174 (2011) 126-133.
[20] Y. Deng, C. Deng, D. Yang, C. Wang, S. Fu, X. Zhang, Preparation, characterization and application of magnetic silica nanoparticle functionalized multi-walled carbon nanotubes, Chem. Commun., (2005) 5548-5550.
[21] W. Zhong, P. Liu, Z. Tang, X. Wu, J. Qiu, Facile approach for superparamagnetic CNT-Fe3O4/polystyrene tricomponent nanocomposite via synergetic dispersion, Industrial & Engineering Chemistry Research, 51 (2012) 12017-12024.
[22] M. Zhang, J. Lu, J.-N. Zhang, Z.-H. Zhang, Magnetic carbon nanotube supported Cu (CoFe 2 O 4/CNT-Cu) catalyst: A sustainable catalyst for the synthesis of 3-nitro-2-arylimidazo [1, 2-a] pyridines, Catalysis Communications, 78 (2016) 26-32.
